# Toward Plasmonic Neuronal Architectures at the Nanometer Scale

**DOI:** 10.1002/nap2.70066

**Published:** 2026-04-09

**Authors:** Christopher G. O. Weiß, Tobias Eul, Emily Kruel, Mario F. Pfeiffer, Bert Lägel, Benjamin Stadtmüller, Martin Aeschlimann

**Affiliations:** ^1^ Department of Physics and Research Center OPTIMAS RPTU University Kaiserslautern‐Landau Kaiserslautern Rhineland‐Palatinate Germany; ^2^ Experimentalphysik II Institute of Physics University of Augsburg Augsburg Bavaria Germany

**Keywords:** dielectric‐loaded SPP waveguides (DLSPPWs), neuromorphic computing, orbital angular momentum (OAM) multiplexing, photoemission electron microscopy (PEEM), plasmonic neural cell, surface plasmon polaritons (SPPs)

## Abstract

We introduce a plasmonic computing platform that unites multiplexed optical inputs, static weighting, and nonlinear activation within a single nanoscale architecture—marking a critical step toward the realization of a plasmonic neural cell. In this approach, the orbital angular momentum (OAM) of light serves as an orthogonal and inherently parallel encoding scheme, enabling distinct optical channels to be directed into individual plasmonic waveguides. The weighting of input signals is governed by precisely engineered nanoscale gaps, whereas the system's nonlinear activation is revealed through two‐photon photoemission, spatially resolved using photoemission electron microscopy (PEEM). By confining electromagnetic fields far below the diffraction limit and minimizing surface plasmon polariton (SPP) propagation lengths, the design mitigates optical absorption losses while fully exploiting the unique field enhancement capabilities of plasmonic nanostructures. This physically integrated platform is compatible with scalable nanofabrication defined through a two‐step electron‐beam lithography (EBL) process and provides a compact, energy‐efficient building block for ultrafast neuromorphic photonic circuits. Together, these results outline a tangible route toward dense plasmonic neural networks capable of performing all‐optical information processing at the native speed of light.

## Graphical abstract

The concept of a plasmonic neural cell is realized through the integration of multiplexed optical inputs, static weighting, and nonlinear readout. The inputs are encoded in the orbital angular momentum of light and coupled into dielectric‐loaded plasmonic waveguides via nanostructured slits. Nanoscale gaps determine the fixed weights, and two‐photon photoemission electron microscopy provides the nonlinear activation signal.

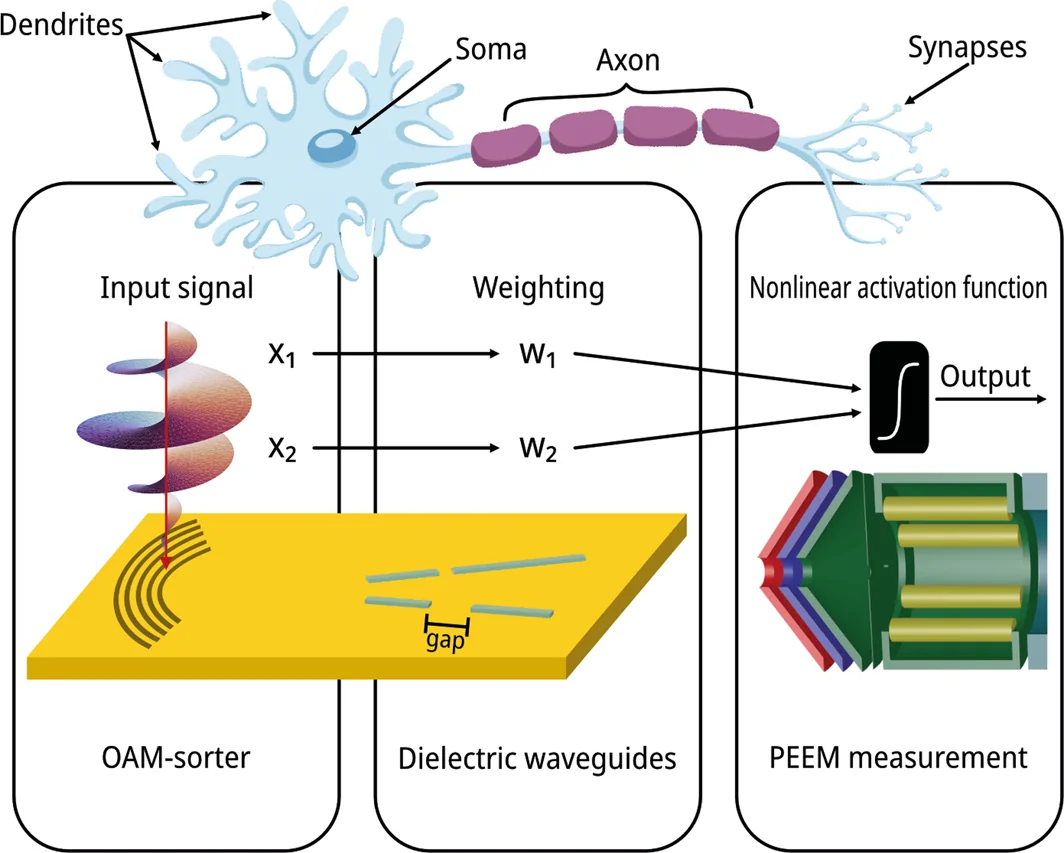

## 1 Introduction

Classical von‐Neumann computers encounter severe energy and speed limitations when simulating large artificial neural networks—the well‐known *von‐Neumann bottleneck* [1]. The continuous drive towards smaller feature sizes and higher integration density therefore necessitates fundamentally different computing paradigms. One longstanding approach involves emulating the operating principles of biological neural networks (BNNs). Since the early days of modern computing, this paradigm has inspired the development of artificial neural networks (ANNs), the majority of which have been implemented as software‐based models. Efforts to realize these systems in physical hardware, whether through electronic or photonic means, have given rise to so‐called neuromorphic architectures, which aim to replicate the structural and functional characteristics of BNNs in a more efficient and scalable form. The term and its vision were established in the 1990s by Carver Mead and others [2, 3]. At their core, neuromorphic neurons rely on three essential building blocks: (i) multiplexing of input channels, (ii) static or dynamic weighting, and (iii) nonlinear evaluation (activation) [4]. The rapid progress of ANNs opens the door to novel hardware‐implemented alternatives that can overcome computing and energy limitations [5, 6].

Photons serve as natural carriers of information in such systems, owing to their ability to propagate at the speed of light, facilitate immense parallelism, and enable low‐energy information transfer [5, 6, 7]. To increase optical data throughput, multiplexing techniques enable the simultaneous transmission of multiple signals within a shared physical medium. Traditional approaches to channel separation exploit degrees of freedom such as wavelength, polarization, and phase. Beyond these, the orbital angular momentum (OAM) of light has emerged as a particularly powerful and versatile degree of freedom. OAM is associated with a helical phase front and is quantized by an integer topological charge (*l*), as first formalized by Allen et al. in 1992. In contrast to polarization, which supports only a small number of orthogonal states, OAM offers a theoretically unbounded set of mutually orthogonal modes, providing a scalable modal basis for high‐capacity optical communication systems [8]. Over the past decade, numerous experimental studies have demonstrated OAM‐based multiplexing with aggregate data rates reaching the terabit‐per‐second regime [9, 10].

Relative to phase‐based encoding schemes, OAM multiplexing does not intrinsically require coherent or interferometric detection, nor does it necessitate precise projection onto a predefined phase basis. As a result, OAM‐based approaches can offer reduced receiver complexity and increased robustness in practical implementations [11].

Wavelength‐division multiplexing, by comparison, is ultimately limited by the available optical bandwidth and typically depends on wavelength‐selective components such as filters or resonators. These components introduce stringent fabrication tolerances and impose ongoing requirements for stabilization and calibration [12]. OAM multiplexing, on the other hand, enables multiple parallel channels at a single operating wavelength, with channel separation achievable through passive, geometry‐defined mode sorting and routing. This characteristic has motivated growing interest in OAM‐based architectures for integrated photonic and plasmonic systems, where reductions in spectral overhead and system complexity are particularly advantageous.

A practical challenge, however, is the mismatch between electronic and photonic length scales: state‐of‐the‐art electronics already operate near the nanometer regime, whereas conventional photonics typically requires micrometer‐scale structures. Plasmonics offers a compelling bridge between electronics and photonics [13]. Propagating surface plasmon polaritons (SPPs), electromagnetic waves bound to metal‐dielectric interfaces, can confine optical fields well below the diffraction limit, enabling subwavelength components and compact guided propagation [14]. Previous studies have demonstrated the implementation of synaptic weighting in plasmonics [15, 16, 17, 18, 19, 20], as well as the implementation of larger networks using SPPs in the microwave regime to realize neural‐network nodes [21, 22, 23]. All these studies highlight that plasmonic networks can offer high integration density, parallel processing, and even direction‐selective functionality.

The next decisive step is now to realize neuromorphic architectures based on SPPs at optical frequencies. These architectures provide much stronger confinement, direct access to light–matter interactions, and compatibility with nanoscale integration. These properties promise compact, ultrafast, and multifunctional neuromorphic networks at the nanoscale. The main trade‐off is metal absorption, which introduces propagation loss. This can be mitigated using waveguide geometries that balance deep subwavelength confinement with acceptable propagation lengths [24, 25, 26]. In this work, we introduce essential functionalities and operating principles that form the basis of a plasmonic neural cell that combines three essential computational steps, namely signal reception, weighted integration, and nonlinear activation, within a single physical platform. As illustrated in Figure 1, the design draws a conceptual analogy to a biological neuron, which receives synaptic inputs via dendrites, integrates them in the soma, and transmits the resulting output along the axon. Similarly, our plasmonic implementation combines these three functional stages in a compact architecture: multiplexed optical inputs, encoded in the orbital angular momentum (OAM) of light, are coupled into dedicated waveguides; the signals are then weighted through precisely engineered nanoscale gaps; and finally, the output undergoes a nonlinear activation process, which is modeled and spatially resolved using nonlinear photoemission electron microscopy (PEEM). This integrated approach enables neuromorphic processing directly in the optical domain, leveraging both the spatial and modal degrees of freedom of structured light.

**FIGURE 1:**
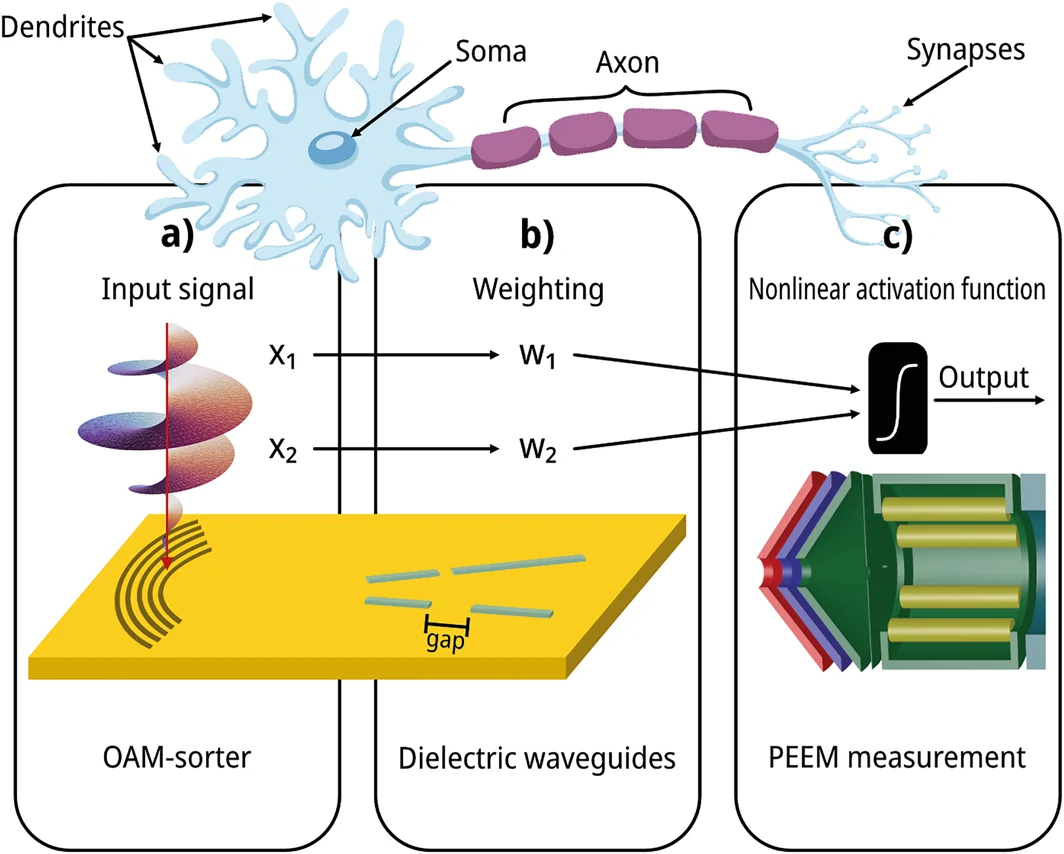
Schematic illustration of the essential building blocks of a plasmonic neural cell concept. Inspired by the operation of a biological neuron (top), our implementation integrates the three key building blocks of a neuromorphic system: (a) multiplexed optical inputs, (b) static weighting, and (c) nonlinear activation. The inputs are encoded in the orbital angular momentum (OAM) of light and coupled into plasmonic dielectric waveguides via nanostructured slits (left). Weighting is implemented by nanoscale gaps in the waveguides (middle), while the nonlinear activation and readout are realized through two‐photon photoemission electron microscopy (PEEM, right).

### 1.1 OAM‐Based Multiplexing of Inputs

We employ an OAM demultiplexer to take a single input stream based on multiple orthogonal signals and route it to many output lines via dedicated plasmonic waveguides (see Figure 1a). Our approach employs electron‐beam lithography (EBL) to define quarter‐circle segment slits on polycrystalline gold, similar to the concepts presented in [27, 28].

Under illumination with OAM light at λ=780nm, the helical phase induces direction‐dependent SPP coupling, producing spatially separated foci that feed distinct waveguides. Earlier related demonstrations include SPP coupling on FIB‐patterned silver waveguides probed by NSOM, albeit controlled via incidence angle rather than OAM [29]. Ren et al. [30] demonstrated OAM sorting on nanowires that enabled the realization of logic gates as well as an integrated on‐chip encryption and decoding system. In addition, a variety of alternative OAM‐multiplexing schemes have been reported in recent years, highlighting the diversity of available approaches [31, 32].

### 1.2 In‐Plane Synaptic Weighting via a Waveguide Gap

The weights are implemented by introducing a nanogap in the plasmonic waveguide (see Figure 1b). The evanescent field across the gap decays exponentially, providing a controllable passive attenuation that realizes the desired static weighting of each input channel. In a subsequent fabrication step, the plasmonic waveguides themselves are also defined by EBL, ensuring precise alignment with the OAM sorter structures. In our layout we use dielectric‐loaded SPP waveguides (DLSPPWs) [33, 34, 35, 36] and leverage the well‐studied trade‐off between confinement and propagation length in gap‐surface‐plasmon geometries [37, 38, 39, 40, 41, 42, 43].

### 1.3 Nonlinear Activation and Readout by PEEM

To simulate the nonlinear activation stage of a neuromorphic system, we perform near‐normal‐incidence (∼4°) PEEM measurements (see *Methods*) to probe the optical near field [44, 45, 46, 47] via the two‐photon photoemission (2PPE) signal at the waveguide terminus (see Figure 1c). The PEEM intensity follows the general scaling formula $I_{\text{PEEM}}\;∼\;{\left|E\right|}^{2n}$, where n denotes the order of the photoemission process. In our case, ${\left|E\right|}^{4}$ for a two‐photon process. The inherently nonlinear dependence of 2PPE on the local SPP intensity serves as a measurement‐induced nonlinear readout mechanism, converting the statically weighted SPP inputs into measurable nonlinear outputs with nanometer spatial precision.

Our approach integrates OAM‐encoded multiplexed inputs, demultiplexing based on OAM‐dependent routing, passive in‐plane weighting and surface‐enhanced measurement‐induced nonlinear readout, where the observed nonlinearity is employed to map the weighted plasmonic near fields to a measurable output. This demonstrates the essential building blocks for a plasmonic neural cell, enabling dense, ultrafast neuromorphic processing with SPPs at optical frequencies. The design exploits the key advantages of plasmonics by confining SPP transport to only the necessary distances, thereby reducing propagation losses while remaining fully compatible with scalable electron‐beam lithography and related nanofabrication techniques.

## 2 Design and Fabrication

For these studies, we mainly used plasmonic neuronal cells consisting of an orbital angular momentum (OAM) sorter and just two plasmonic waveguides, each of which incorporates a nanoslit of a specific size. The optical microscope image in Figure 2a shows the overall layout of the structure, while Figure 2b provides a schematic illustration of the fabricated nanostructures on a polycrystalline gold island. The OAM demultiplexer (sorter) consists of concentric trench segments milled into a polycrystalline gold island deposited on a silicon substrate. The concentric trench segments geometry features quarter‐circle arcs arranged in five successive sections, forming a grating designed to enhance coupling efficiency for surface plasmon polaritons (SPPs). Depending on the orbital angular momentum of the incoming light, the structure generates a spatially distinct plasmonic focus at the geometric center of the trench segments. Starting from the focal points of these concentric trench segments, polymer (PMMA) waveguides are patterned on top of the polycrystalline gold island.

**FIGURE 2:**
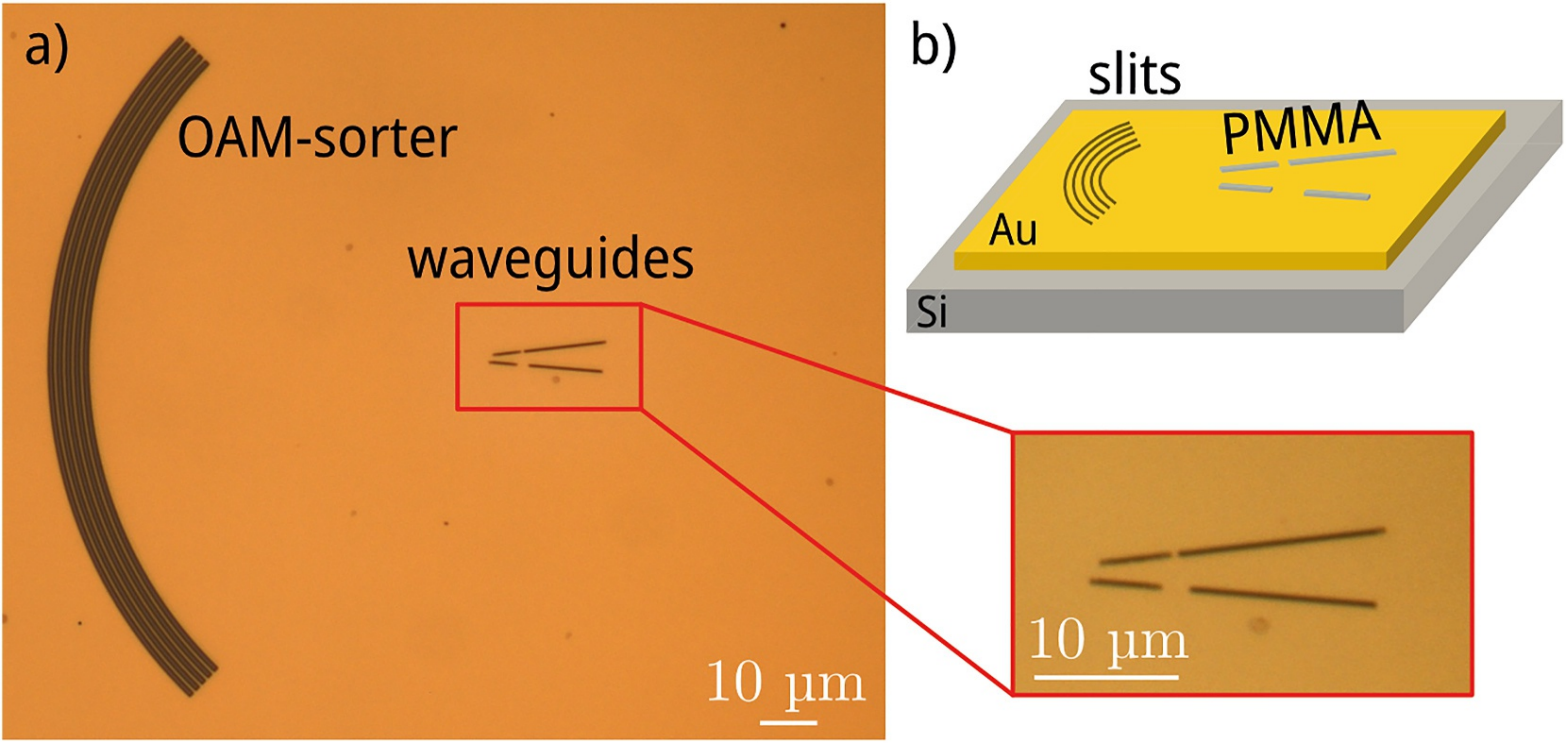
(a) Optical microscope image showing the central parts of our essential building blocks of a plasmonic neural cell concept, consisting of an OAM sorter and two adjacent waveguides with defined gaps. (b) Schematic illustration of the nanostructures fabricated on a polycrystalline gold island on top of a Si substrate.

### 2.1 Optimization by FDTD Simulations

In order to identify the most efficient implementation of both the OAM sorter and the plasmonic waveguides, we first carried out finite‐difference time‐domain (FDTD) simulations (see *Methods*) on scaled‐down model structures. These simulations guided the choice of geometry and material for the experimental realization. Based on the simulation results, the final structure was fabricated using a two‐step electron‐beam lithography (EBL) process, as detailed in the following sections. The sorter was implemented either as (i) trenches structured into the gold film or ridges made of (ii) PMMA or (iii) SiO_2_ deposited on top of the gold. The calculated near‐field intensity distributions of the out‐of‐plane field component ${\left|E_{z}\right|}^{2}$ are shown in Figure 3a. Figure 3b summarizes the corresponding normalized maximum values of ${\left|E_{z}\right|}^{2}$ obtained from the simulated field distributions. The normalization was performed with respect to the peak value of the trench structure, determined from the *x*‐ and *y*‐profiles of ${\left|E_{z}\right|}^{2}$ for the trench, PMMA, and SiO_2_ configurations.

**FIGURE 3:**
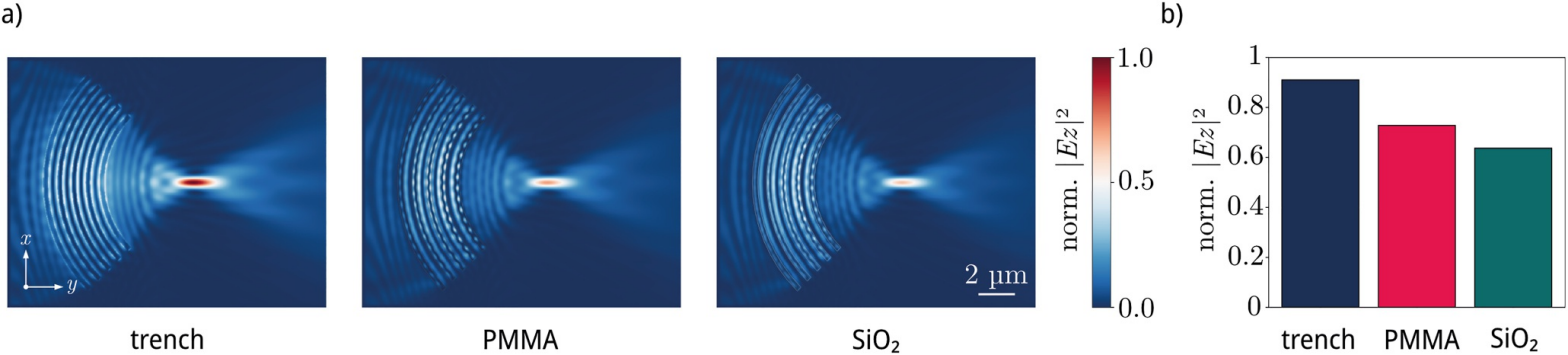
Finite‐difference time‐domain (FDTD) simulations comparing different implementations of the OAM sorter. (a) Calculated near‐field intensity distributions ${\left|E_{z}\right|}^{2}$ for a trench geometry (left), a PMMA ridge (middle), and a SiO_2_ ridge (right). (b) Corresponding efficiencies extracted from the simulations, showing that the trench geometry provides the highest coupling efficiency into surface plasmon polaritons.

The comparison clearly shows that the trench geometry provides a stronger coupling of the incident OAM beam into surface plasmon polaritons than the ridge‐type designs. Because efficient SPP generation is crucial for the functionality of the device, these findings guided our decision to implement the OAM sorter as a set of trenches fabricated in the gold film. The dimensions of the trenches are optimized for efficient SPP excitation at the illumination wavelength of $\lambda_{L}=780\;\text{nm}$ [48]. Each trench has a width of 950 nm, and the trench period corresponds to twice the plasmon wavelength $\lambda_{\text{SPP}}=762\;\text{nm}$, consistent with the general condition that maximum grating–coupling efficiency is achieved when the slit separation is an integer multiple of the plasmon wavelength [49, 50].

In a second step, we applied FDTD simulations to optimize the design of the plasmonic waveguides. Here, we compared three possible realizations: (i) trenches directly structured in the gold film, (ii) ridge waveguides made of PMMA, and (iii) ridge waveguides made of SiO_2_. Figure 4a illustrates the simulated near‐field distributions of ${\left|E_{z}\right|}^{2}$ for all three configurations, while Figure 4b shows the corresponding line profiles o ${\left|E_{z}\right|}^{2}$ along the propagation axis. The simulations reveal that trench‐type waveguides support almost no guided signal, confirming that this geometry is unsuitable for efficient plasmon transport. In contrast, both PMMA and SiO_2_ ridge waveguides exhibit nearly identical transmission characteristics, with strong confinement and propagation of the plasmonic mode.

**FIGURE 4:**
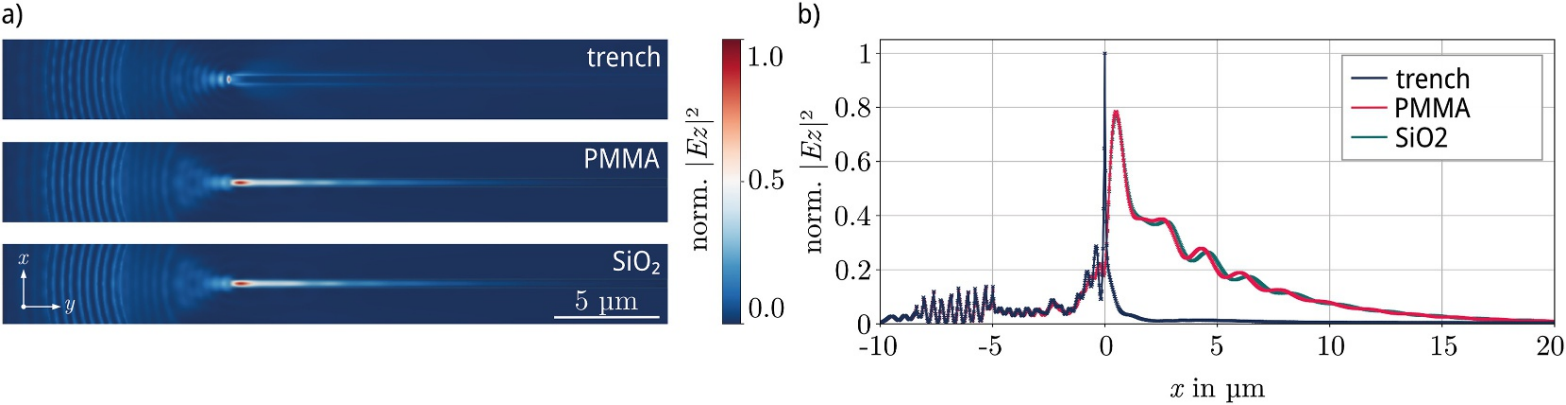
Finite‐difference time‐domain (FDTD) simulations of different waveguide implementations. (a) Near‐field intensity distributions ${\left|E_{z}\right|}^{2}$ for a trench waveguide (top), a PMMA ridge (middle), and a SiO_2_ ridge (bottom). (b) Line profiles of ${\left|E_{z}\right|}^{2}$ along the propagation direction, demonstrating that the trench waveguide supports almost no guided signal, while PMMA and SiO_2_ show comparable transmission efficiencies.

Whereas SiO_2_ and PMMA show comparable optical performance, the choice between the two materials is determined by fabrication considerations. PMMA offers the clear advantage of a simpler process flow, since it can be directly defined by electron‐beam lithography without the need for an additional evaporation step. We therefore selected PMMA as the material for the plasmonic waveguides in the final device design.

The PMMA waveguides width and height were also optimized by FDTD simulations (see Supporting Information S1, Section S1). The simulations revealed an optimal geometry with a width of 300 nm and a height of 180 nm, providing efficient guiding while minimizing cross‐talks. In the fabricated devices, the waveguides were realized with a width of 300 nm, in agreement with the simulated optimum, but with a reduced height of 45 nm due to fabrication constraints. This deviation leaves room for further improvement, as taller waveguides would enhance guiding efficiency.

### 2.2 Fabrication

All fabrication steps, including both electron‐beam lithography processes and subsequent electron‐beam evaporation, were carried out at the Nanostructuring Center (NSC) of the RPTU University Kaiserslautern‐Landau. We investigate two samples in this work. The fabrication workflow is identical for both and differs only in the configuration of the PMMA waveguides. The first sample features continuous waveguides (no gap) with a length of 100 μm. The second sample incorporates synaptic gaps: the upper waveguide features a 0.5 μm gap and the lower waveguide a 2 μm gap. The waveguides in the second sample have a total length of 20 μm, and the gaps are positioned 5 μm from the left waveguide edge (sorter focal point). For both samples, the PMMA waveguides were fabricated with a width of 300 nm and a height of 45 nm.

In the first electron beam lithography (EBL) step, the Si substrate is spin‐coated with a positive‐tone resist (CSAR 62–AR‐P 6200.09, Allresist GmbH). Because CSAR is a positive resist, the exposed regions are removed during development, whereas the unexposed areas remain on the substrate. A 400 μm × 400 μm square is then exposed, whereas the ring‐segment pattern defining the OAM sorter is intentionally left unexposed. After development, only the sorter structure remains as CSAR ridges. The sample is then cleaned in an O_2_ plasma to remove resist residues. Subsequently, a 10 nm Ti adhesion layer followed by 140 nm Au are deposited by electron‐beam evaporation, covering the remaining CSAR ridges with the deposited metal layer. During the final lift‐off in acetone, the CSAR ridges (together with the metal on top) are removed, leaving trenches with the ring‐segment geometry that form the OAM sorter in the metal film. Finally, the sample is cleaned in isopropanol and dried under N_2_ gas. Additionally, alignment crosses were patterned during the first EBL step to enable precise positioning of the plasmonic waveguides in the next fabrication step.

In a second step, a PMMA resist layer (AR‐P 672.02–950k dissolved at 2% in anisole, Allresist GmbH) is applied and patterned by EBL in a negative‐tone process, such that the exposed regions remain after development and define the plasmonic waveguides. The gaps are defined simply by leaving the corresponding waveguide segment unexposed during EBL. After exposure, the sample is developed in acetone such that the unexposed PMMA is removed and the intentionally omitted segment dissolves and forms the gap. Alignment is performed with respect to the gold crosses written in the first step, ensuring precise placement of the waveguides relative to the sorter's focal spots. As the sorter radius r in our design scales with the absolute OAM order |l| [51], we fabricated several variants on the same substrate to match the beam diameters of the targeted modes: $\left|l\right|=2\;(r=50\;µm)$, |l|=4(r=50µm), |l|=6(r=70µm), and |l|=8(r=75µm).

As a final preparation step prior to the PEEM measurements, the samples were coated with a sub‐monolayer of cesium. This treatment reduces the work function and thereby enables a two‐photon photoemission (2PPE) process at the excitation wavelength of $\lambda_{L}=780\;\text{nm}$.

## 3 Results and Discussion

For this study, we use a photoemission electron microscope (PEEM) which provides direct real‐space access to the spatial distribution of photoemitted electrons from a sample surface with nanometer‐scale resolution (see *Methods*).

### 3.1 Multiplexing With Orbital Angular Momentum

Firstly, we optimize the splitting of the focal spots as a function of the orbital angular momentum (OAM), without coupling to plasmonic waveguides. The OAM sorter provides the physical basis for demultiplexing, as it allows different OAM channels to be separated into distinct spatial modes. Figure 5 presents a schematic illustration of the experimental PEEM setup. The photoelectrons emitted from the sample are imaged onto a detector, providing real‐space information. The left panel shows a representative PEEM image of the sample illuminated with a mercury lamp.

**FIGURE 5:**
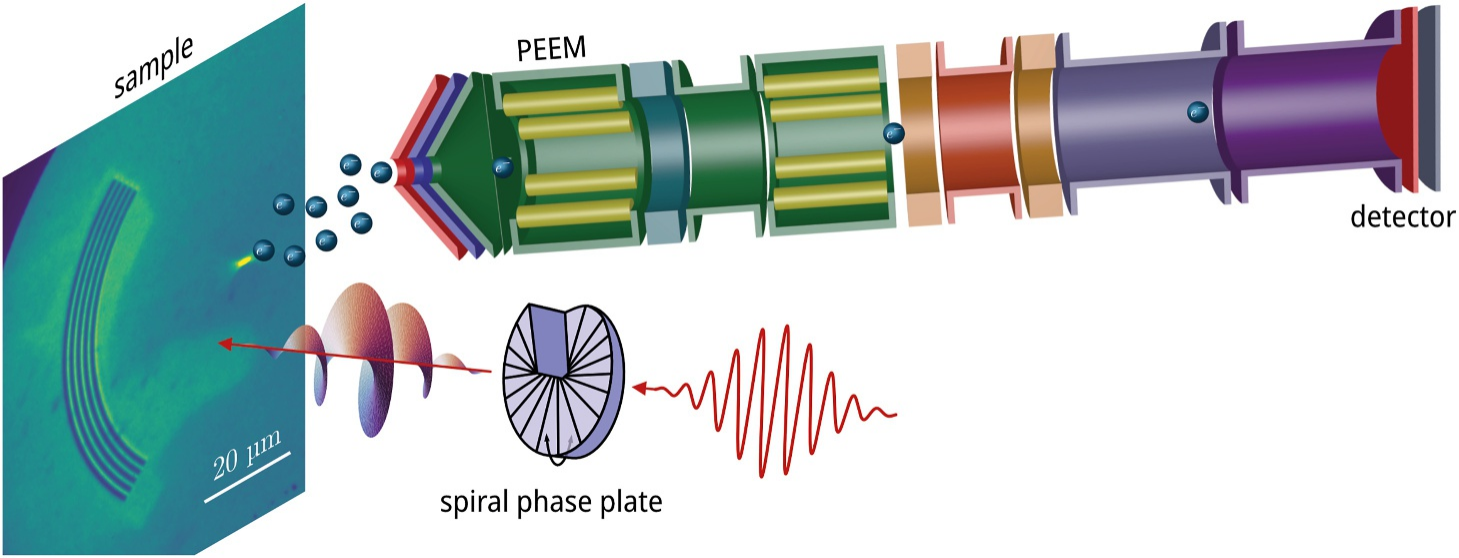
Schematic illustration of the experimental approach. A nanofabricated OAM sorter (false‐color PEEM image on the left, taken under Hg‐lamp illumination) converts incoming optical vortex modes into surface plasmon polaritons. The excited plasmonic near fields are investigated with photoemission electron microscopy (PEEM) using ultrashort laser pulses at near‐normal incidence. A spiral phase plate imparts orbital angular momentum to the pulses before they interact with the structure. The emitted electrons are collected and projected through an electron‐optical lens system onto a detector.

Excitation is carried out with ultrashort, linearly polarized laser pulses under near‐normal incidence. Passing the laser beam through a spiral phase plate (VPP‐m780 model from VIAVI Solutions) generates light carrying OAM. The plate is designed such that OAM orders from l=1 to l=8 are spatially separated within a single optical element. By flipping the spiral phase plate, the sign of the OAM can be inverted. The generated light forms a donut mode with a characteristic radius. We extend this investigation by including an additional degree of freedom: the spin angular momentum (SAM). A quarter‐wave plate placed after the spiral phase plate generates left‐ or right‐circularly polarized light. The total angular momentum j of the light is then given by j=l+s. We measure all accessible even OAM orders (l=±2,±4,±6,±8) combined with spin states σ=0,±1. Different nanostructures are used for different OAM values to account for the varying donut beam diameters.

Figure 6 summarizes the results, showing the linear dependence of the spatial separation $d_{y}$ on the total angular momentum j of the incident light. Each color marker represents a single measurement. The focal spots in the PEEM images are fitted with two‐dimensional Gaussian functions, and their relative positions are referenced to the case l=0 and *s* =0.

**FIGURE 6:**
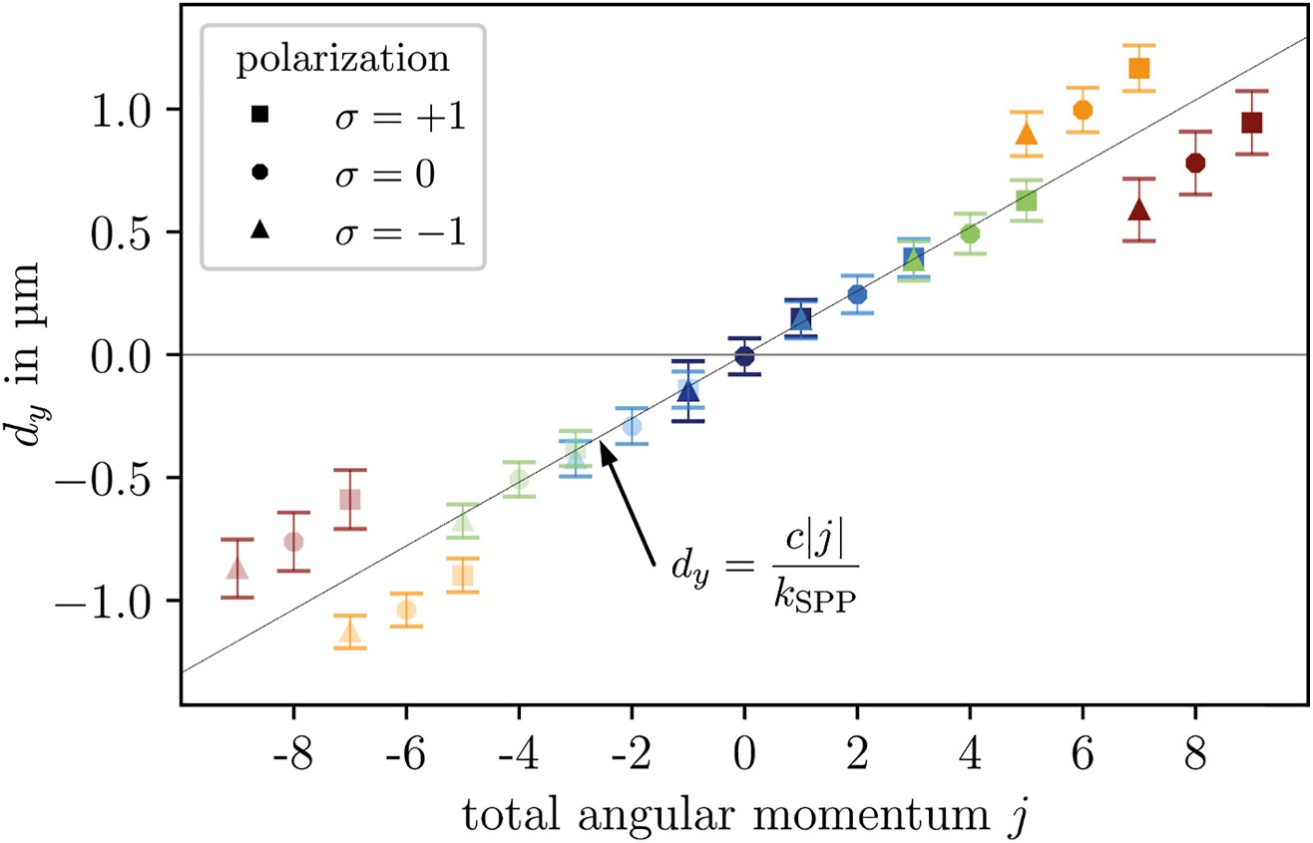
Measured transverse displacement $d_{y}$ as a function of the total angular momentum j=l+σ. Symbols denote different incident polarizations (σ=+1,0,−1). The experimental data (colored markers) follow the linear scaling law $d_{y}=c\left|j\right|/k_{\text{SPP}}$ (gray line), demonstrating the expected proportionality between the plasmonic focal shift and the incident total angular momentum.

A comparison with the theoretical prediction from Ref. [28] shows reliable agreement within experimental uncertainty. Only the measurements for l=±6 deviate to larger values, whereas those for l=±8 fall below the predicted trend. This discrepancy may be attributed to a slight misalignment of the spiral phase plate. The theoretical focal point positions are described by the following equation:

$$d_{y}=\frac{c\left|j\right|}{k_{\text{SPP}}},$$

with *c* = 1.067 as a constant, |j| the absolute value of angular momentum, and $k_{\text{SPP}}$ the surface plasmon polariton wavevector. These findings confirm that our OAM sorter functions reliably and that PEEM provides a suitable method to resolve the corresponding focal spots.

Secondly, we verify that the OAM‐dependent focal spots can indeed couple into the plasmonic waveguides. Figure 7 shows three representative PEEM images of the same structure. Figure 7a shows an overview of the two waveguides illuminated by a mercury lamp. Figure 7b presents laser excitation with OAM orders l=+8 and Figure 7c with l=−8. In both cases, the focal spot is guided into a distinct waveguide, demonstrating a clear channel separation for opposite OAM values. This initial test without any gap in the waveguides confirms that efficient coupling is achieved.

**FIGURE 7:**
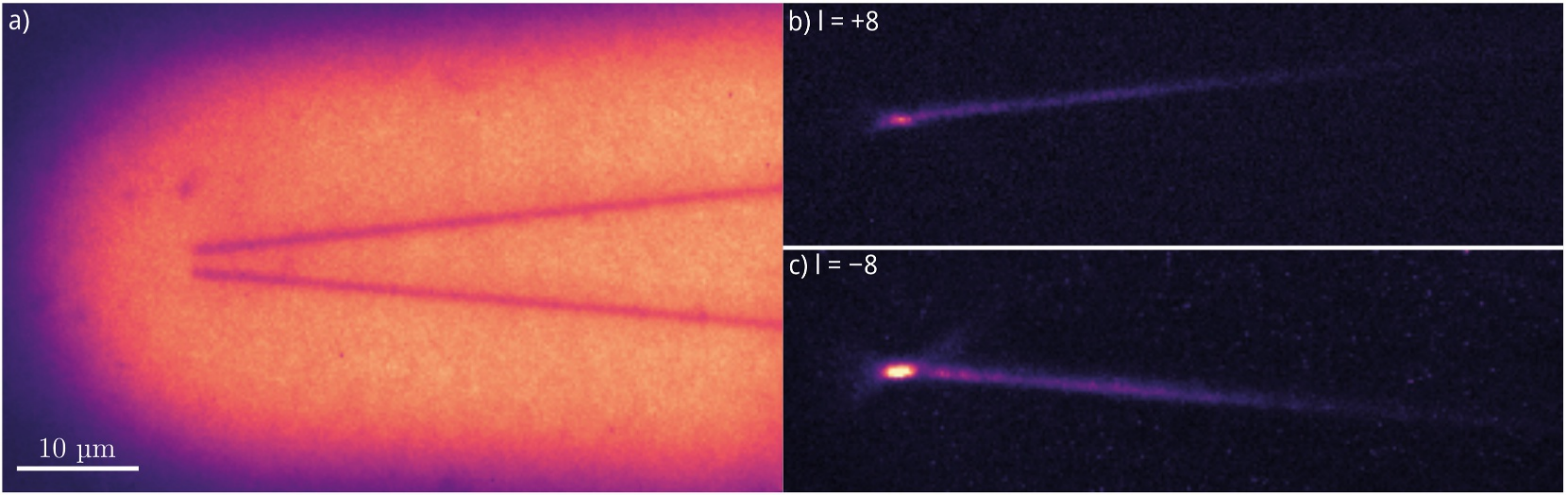
PEEM images of the plasmonic waveguide structure. (a) Illumination with a mercury lamp. (b, c) Excitation with ultrashort laser pulses carrying orbital angular momentum (OAM). For (b) l=+8 and (c) l=−8, the focal spot couples into different waveguides. In this initial test without a gap, the measurements confirm that distinct OAM orders are efficiently coupled into separate channels.

### 3.2 Weighting Functionality of Plasmons

Third, based on these results, we proceed to study the weighting functionality of plasmons during their propagation in the waveguides. In the neuron model as discussed above, the separation of the input signals by the OAM sorter corresponds to the function of dendrites in biological neurons. Weightings can be used to specifically amplify or attenuate signal components. This not only controls information processing but also enables the learning process in artificial neural networks: changes in the weightings correspond to the adaptation to training data.

In this first approach, we examine only static weighting. Such fixed weights offer the advantage for specialized applications that the functionality can be optimized once and then executed reproducibly with low energy consumption. They thus contribute to the efficiency and simplification of the architecture. The implementation chosen here relies on a controlled attenuation of the signal achieved by introducing a gap in the dielectric waveguide. Figure 8 (left) illustrates this concept, showing the schematic and geometric dimensions of the simulated PMMA waveguide on gold. The size of the gap determines the strength of the attenuation. As the gap length increases, the divergence of the transmitted beam increases, so that a growing proportion of the optical energy is no longer coupled into the modes of the opposite waveguide but is emitted as radiation loss into the surrounding space.

**FIGURE 8:**
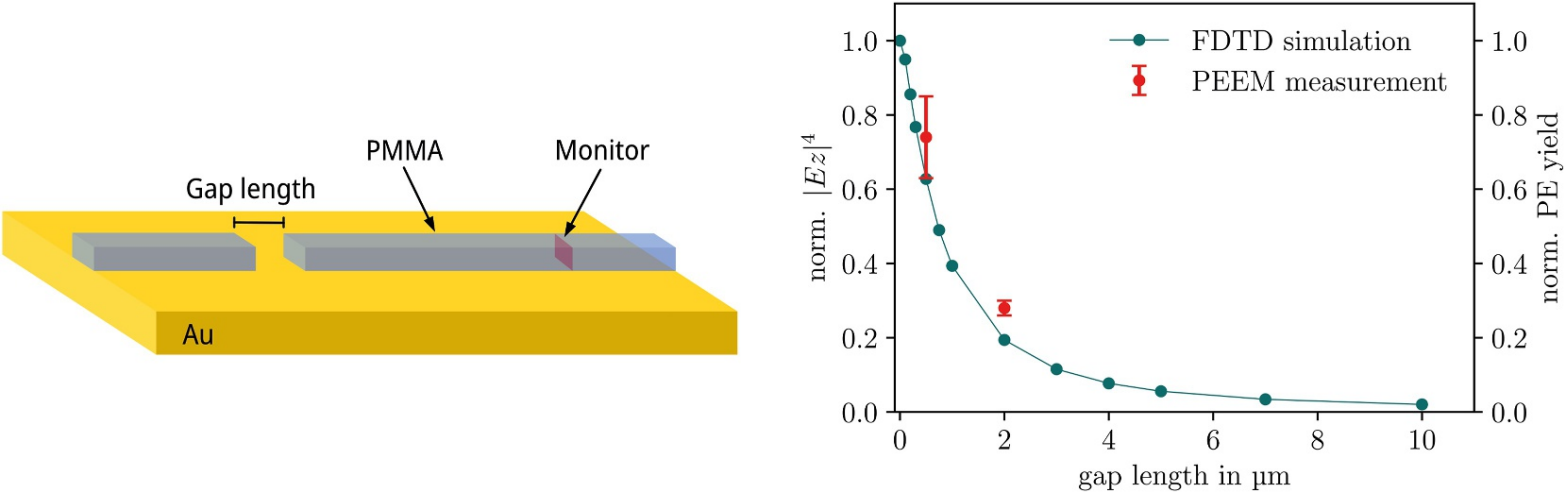
(left) Schematic drawing of the geometric dimensions of the simulated PMMA waveguide on gold. (right) Gap‐length dependence of the normalized transmitted near‐field metric ${\left|E_{z}\right|}^{4}$ obtained from FDTD (green) and the corresponding normalized PEEM photoemission (PE) yield (red).

To illustrate the basic principle of operation, an FDTD simulation is first performed. The transmitted signal was compared as a function of the gap size by means of an electric field monitor placed behind the gap to record the remaining mode intensity inside the waveguide.

The green points in Figure 8 (right) represent the normalized fourth power of the simulated field distribution ${\left|E\right|}^{4}$, where the normalization is performed with respect to the corresponding reference simulation without a gap. In contrast, the red points show the normalized experimentally measured 2PPE photoemission (PE) yield, where the PEEM signal is normalized to the photoemission yield measured at the gap position. Because the 2PPE‐PEEM intensity scales nonlinearly with the local electric field as ${\left|E\right|}^{4}$, the normalized experimental signal can be directly compared to the normalized ${\left|E\right|}^{4}$ values obtained from the FDTD simulations.

The signal strength decreases exponentially with increasing gap size within the μm range. Therefore, the FDTD simulation provides an estimation of the gap size required for a specific weighting. To determine the experimental uncertainty, regions of interest (ROIs) were defined inside the gap and in a background area behind the waveguide. The error bars of the measured data were obtained from the statistical fluctuations of the photoelectron count rates, quantified by the standard deviation. In addition, the sensitivity to the exact ROI definition was assessed by repeating the analysis while varying the ROIs by a few pixels (single‐digit pixel shifts). The resulting spread was combined with the statistical uncertainty to yield the total error using Gaussian error propagation.

For these experiments, we restrict ourselves to even OAM orders without additional spin angular momentum. Figure 9 shows a representative PEEM image of the double waveguide structure excited with l=±8. In Figure 9a, the plasmonic near‐field enhancement couples exclusively into the upper waveguide for l=+8, where it crosses the 0.5 μm gap and yields a distinct emission spot at the waveguide end. This bright spot at the waveguide terminus is attributed to outcoupled light that generates photoelectrons via a two‐photon photoemission (2PPE) process [52]. In Figure 9b, excitation with l=−8 couples the signal into the lower waveguide, where it crosses the 2 μm gap and still produces an emission feature at the waveguide end, although with noticeably reduced intensity. Within the gap region, a bright emission is observed, which we attribute to an enhanced photoelectron yield caused by strong near‐field enhancement at the gap location. This interpretation is supported by our FDTD simulations (see Supporting Information S1, Section S2), performed for a waveguide geometry comparable to the experiment (width 300 nm, height 50 nm) and a gap length of 0.5 μm, which show a pronounced local field‐intensity enhancement at the gap. The corresponding experimentally obtained normalized intensities of these distinct bright spots are plotted as red points in Figure 8 (right) and are in fairly good agreement with the FDTD simulation.

**FIGURE 9:**
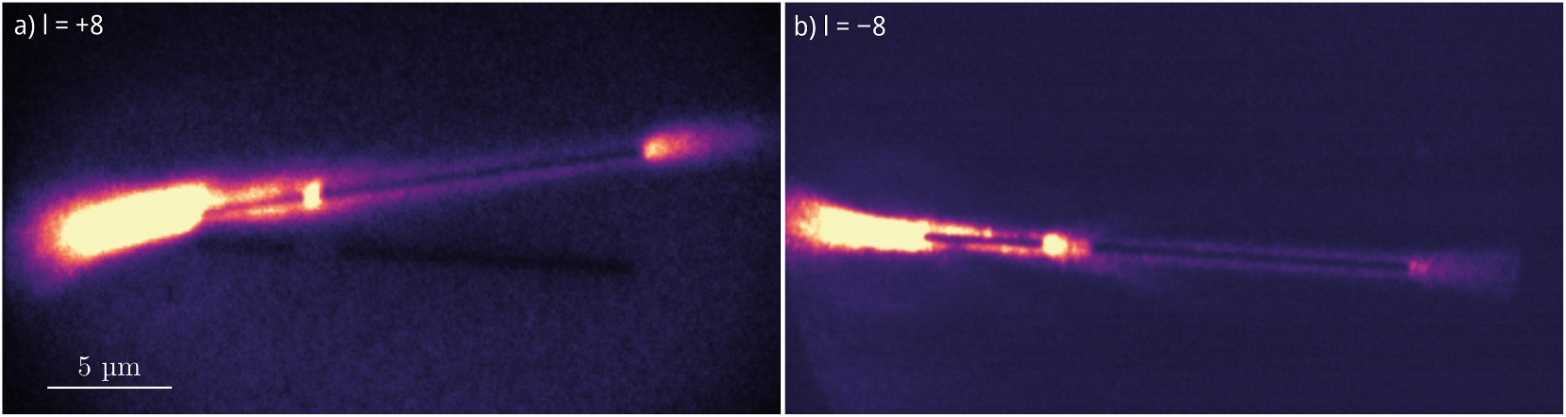
PEEM image of the double waveguide structure excited with OAM states l=±8. (a) For l=+8 the SPPs couple into the upper waveguide and propagate across the 0.5 μm gap. A bright emission spot at the waveguide end indicates outcoupling that generates photoelectrons. (b) For l=−8, the SPPs analogously couple into the lower waveguide and propagate across the 2 μm gap. A photoelectron‐emission spot is also present at the waveguide end, but with reduced intensity.

Overall, the results confirm that a targeted attenuation of the plasmonic signal can be achieved by introducing a gap in the waveguide. Although the 0.5 μm gap in these measurements is slightly obscured by experimental uncertainties, the 2 μm gap leads to a clear and reproducible weighting. This allows controlled static weighting to be integrated as a functional element into the concept of the plasmonic neuromorphic cell.

## 4 Conclusions and Outlook

In summary, this work represents a first step toward the realization of a neuromorphic plasmonic network on a nm scale. We establish the fundamental operational framework of a plasmonic neural cell, an integrated nanophotonic element that consolidates signal reception, weighted integration, and measurement‐induced nonlinear activation within a single physically unified platform. The proposed architecture draws a direct analogy to the organization and function of a biological neuron, which receives multiple synaptic inputs through dendritic branches, performs signal integration within the soma, and subsequently propagates the resultant output along the axon. In our plasmonic implementation, we emulate key neuronal functions within a compact and fully plasmonic structure. Multiple optical inputs, each encoded in distinct orbital angular momentum (OAM) states of light, are simultaneously coupled into separated plasmonic waveguides that act as input channels. Within the waveguides, the signals experience amplitude weighting governed by precisely engineered nanoscale gaps, which determine the strength of plasmonic interactions and thereby define the effective synaptic weights. The weighted optical fields are then mapped to a nonlinear output via plasmon‐enhanced two‐photon photoemission, which in the present work serves as a nonlinear readout mechanism. This nonlinear response can be spatially resolved and characterized quantitatively using photoemission electron microscopy (PEEM), which enables direct visualization of the activation dynamics at the nanoscale.

As an outlook, spatial light modulators (SLMs) could be employed to generate perfect vector‐vortex beams [53] whose mode diameter remains independent of the OAM order. This would allow OAM beams of different orders to address the same plasmonic structure under identical excitation conditions, thereby significantly enhancing the flexibility and scalability of OAM‐based plasmonic architectures.

In our work, 2PPE PEEM was just used to simulate the nonlinear activation stage of a neuromorphic system. For practical neuromorphic networks, a nonlinear functionality could be embedded directly between cells. One promising approach is to integrate hybrid material systems, such as graphene/PMMA junctions [54], which exhibit a sigmoid‐type activation response and would thereby provide an intrinsic on‐chip nonlinear computational element.

Although the present experimental demonstration is intentionally restricted to a minimal structure comprising two plasmonic waveguides, the underlying OAM‐multiplexing/sorting principle is not limited to two channels: in principle, multiple waveguides can be placed at the distinct OAM‐dependent focal positions to increase the input dimensionality and information density. In this context, Mei et al. [27] note that the maximum practically sortable OAM range of the semi‐ring nanoslit concept is limited only by SPP propagation losses. Representative propagation lengths on gold are on the order of 10–80 μm at wavelengths λ=600–750nm [55], while they can reach ∼10 mm in the mid‐IR (λ=10.6µm) [56]. Importantly, scaling to higher topological charges |l| and to more waveguides also requires that the spatial separation between neighboring OAM focal spots remains sufficiently large relative to the guided‐mode extent to avoid unintended excitation (cross‐talk) of adjacent waveguides. This would enable the transition from a proof of concept to scalable plasmonic neuromorphic networks.

Representative propagation metrics help to quantify the confinement–loss trade‐off associated with plasmonic waveguides. Long‐range plasmonic waveguides have been reported with losses of a few up to ∼10 dB/mm in the visible/NIR, corresponding to 1/e intensity propagation lengths on the order of several hundred micrometers, and with millimeter‐scale propagation at telecom wavelengths [57]. For the tens‐of‐micrometers propagation distances employed in our present devices, such losses would imply only a modest attenuation, suggesting that the current end‐to‐end efficiency is likely dominated by the coupling efficiency at the OAM sorter‐waveguide interface rather than by propagation loss along the waveguides themselves. A dedicated quantitative assessment and optimization of both propagation loss and coupling efficiency in our specific geometry remains an important direction for future work.

In addition, signal weighting is a crucial aspect of learning‐capable architectures. In our present implementation, the weighting mechanism is statically defined by nanoscale gaps in the waveguides and cannot therefore be reconfigured. Alternative strategies have already been proposed to achieve dynamic weighting. For instance [58], showed that an LRSPP mode propagating in the *x*‐direction across a waveguide slit can be actively modulated by an optical control signal incident in the *y*‐direction. This control beam excites plasmonic edge modes at the slit, which in turn influence the propagation of the LRSPP mode. Such concepts provide promising pathways toward dynamically reconfigurable synaptic weights and, ultimately, adaptive plasmonic neural networks.

Altogether, our results establish the essential building blocks of a plasmonic neural cell and outline clear routes toward adaptive large‐scale plasmonic neuromorphic networks. With further advances in nanofabrication and dynamic control concepts, plasmonics may thus provide a viable platform for ultrafast, nanoscale, all‐optical information processing.

## 5 Methods

### 5.1 Finite‐Difference Time‐Domain Simulations (FDTD)

Metasurfaces were designed and analyzed using the FDTD method implemented in *Tidy3D (Flexcompute Inc.)* [59]. The computational domain was terminated by perfectly matched layers (PML) on all sides to suppress spurious reflections and to emulate an open‐space environment, with vacuum as the embedding medium. The overall simulation domain was chosen to be approximately one order of magnitude larger than the investigated structure to minimize boundary effects. Large‐scale features without near‐field relevance were down‐scaled to reduce computational cost. The spatial discretization was determined using *Tidy3D*'s autogrid feature, with the minimum number of grid points per wavelength set to 20. The mesh was further refined locally in regions of high field gradients to ensure numerical accuracy while maintaining reasonable computational cost.

The simulated structures consisted of a silicon substrate with a thickness of 2 μm and a gold layer of 140 nm on top. The dielectric properties of all materials were taken from the *Tidy3D* material library. The dielectric properties of all materials were taken from the *Tidy3D* material library, using dielectric functions for gold from [60], for silicon from [61], and for PMMA and SiO_2_ from [62].

The simulations were run for a total duration of 3 ps, with a shutoff condition applied that automatically terminated the simulation when the ratio of the instantaneous integrated electric‐field magnitude to its maximum value fell below $1\times 10^{-5}$, thus avoiding unnecessary computation once all fields had decayed. Excitation was provided by a Gaussian beam with a central wavelength of 780 nm and a pulse width of 40 fs. For the gap configuration, an additional FDTD simulation employing a guided‐mode source was conducted. The mode was launched by the same Gaussian pulse, and the plasmonic waveguide was modeled with a width of 300 nm and a height of 100 nm. The gap was positioned 1 μm downstream of the mode source, whereas field monitors were placed 3 μm beyond the gap for all gap widths.

### 5.2 Laser Setup

The PEEM measurements are driven by a titanium–sapphire (Ti:Sa) laser oscillator with a central wavelength of $\lambda_{L}=780\;\text{nm}$ (1.6 eV). The source provides an average optical power of 650 mW, delivering pulses with an energy of 8 nJ at a repetition rate of 80 MHz and a pulse duration of τ=40fs.

### 5.3 Photoemission Electron Microscopy (PEEM)

For this study, we use a photoemission electron microscope (PEEM) from FOCUS GmbH. The PEEM technique provides direct real‐space access to the spatial distribution of photoemitted electrons from a sample surface with nanometer‐scale resolution, with a lateral resolution of approximately 50 nm under the operating conditions used here. By employing an electrostatic lens system, the emitted electrons are accelerated by an extractor voltage of 9 kV and projected onto a phosphor screen, where they are detected with a CCD camera. In this way, PEEM combines high surface sensitivity with the ability to capture two‐dimensional electron emission patterns from the entire field of view in a single exposure. This capability enables to map optical near fields and localized electronic excitations. This makes PEEM a particularly powerful tool for investigating plasmonic nanostructures, as the local photoemission intensity scales nonlinearly with the near‐field strength. In our experiments, this capability enables us to determine how structured optical excitations, such as light carrying orbital angular momentum (OAM), translate into spatially separated electron emission patterns on the surface of a nanostructure, which can then be coupled into plasmonic waveguides.

## Author Contributions

**Christopher G. O. Weiß:** writing – original draft, conceptualization, visualization, investigation, writing – review and editing, methodology, project administration. **Tobias Eul:** conceptualization, methodology, investigation, supervision, project administration, writing – review & editing. **Emily Kruel:** conceptualization, methodology, software, investigation, visualization, writing – review & editing. **Mario F. Pfeiffer:** conceptualization, software, investigation, visualization, writing – review & editing. **Bert Lägel:** methodology, writing – review & editing. **Benjamin Stadtmüller:** conceptualization, supervision, project administration, resources, writing – review & editing. **Martin Aeschlimann:** conceptualization, supervision, project administration, resources, writing – review & editing.

## Funding

The authors have nothing to report.

## Conflicts of Interest

The authors declare no conflicts of interest.

## Supporting information


Supporting Information S1

## Data Availability

The datasets generated and/or analyzed during the current study are available from the corresponding author upon reasonable request.
