# Supplementary material for: Toward Plasmonic Neuronal Architectures at the Nanometer Scale
Source: Nanophotonics. 2026 Apr 9;15(7):e70066. doi: 10.1002/nap2.70066 (PMC13642609; doi:10.1002/nap2.70066)
Supplement: Supplementary file 1 [file NAP2-15-e70066-s001.docx]

# Supplementary Material

## Toward Plasmonic Neuronal Architectures at the Nanometer Scale

Christopher G. O. Weiß^1,*^, Tobias Eul^2^, Emily Kruel^1^, Mario F. Pfeiffer^1^, Bert Lägel^1^, Benjamin Stadtmüller^2^, and Martin Aeschlimann^1^

^1^ Department of Physics and Research Center OPTIMAS, RPTU University of Kaiserslautern-Landau, 67663 Kaiserslautern, Germany

^2^ Experimentalphysik II, Institute of Physics, University of Augsburg, 86159 Augsburg, Germany

^*^ Corresponding author: weissc@rptu.de

### S1. Structural optimization simulation

Structural optimization simulations were conducted to determine the most suitable waveguide width and height. This optimization is based on an eigenmode analysis of the waveguide cross-section for PMMA ridge waveguides of varying width and height on a 140-nm-thick gold film. The FDTD simulations were performed with Tidy3D [1] and are presented as a heat map in Figure S1. The waveguide dimensions were swept parametrically and the maximum value of the cross-sectional ${|E_{z}|}^{2}$ field component of the fundamental TM_00_ mode inside the waveguide was extracted and plotted, normalized to the global maximum within the full parameter set. For small cross-sections, no propagating guided mode is supported, as indicated by the black region in the lower-left corner. Notably, for a width of $w=300 \mathrm{nm}$, propagating modes appear for heights $h\geq40 \mathrm{nm}$, and the field intensity inside the waveguide increases with increasing height. The waveguides were fabricated with a width of 300 nm, but due to fabrication constraints, the height used in the experiments is limited to $h=45 \mathrm{nm}$. The coupling and guiding remain functional, while the simulation indicates further potential for improved confinement and efficiency by increasing the waveguide height within the single-mode regime.

**Figure S1:** FDTD simulations of PMMA ridge waveguides on a 140-nm Au film for parametrically varied width and height. The map shows the maximum ${|E_{z}|}^{2}$ inside the waveguide, normalized to the global maximum. For small cross-sections, no guided mode is supported.

### S2. Field intensity distribution along the waveguide

To support the interpretation of the localized bright emission observed in PEEM, we performed a three-dimensional finite-difference time-domain (FDTD) simulation using Tidy3D [1]. The model consists of a PMMA ridge waveguide with a width of 300 nm and a height of 50 nm, placed on a 140 nm thick gold (Au) film. A gap is introduced into the waveguide with a length of 0.5 µm along the propagation direction.

The structure is excited such that a guided plasmonic mode is launched into the PMMA waveguide, and the resulting electromagnetic field distribution is evaluated in the vicinity of the structure. We analyze the normalized electric-field intensity ${|E_{z}|}^{2}$ in a plane close to the waveguide, as shown in Figure S2, to visualize how the field evolves along the waveguide and across the gap. The white boxes mark the area of the waveguide.

The simulation reveals a pronounced near-field intensity hotspot at the gap location, while the intact waveguide section exhibits a weaker, distributed field intensity. The gap acts as a strong discontinuity for the guided mode, leading to local field concentration and enhanced ${|E_{z}|}^{2}$. This strong near-field enhancement at the gap provides a straightforward explanation for the experimentally observed bright signal in PEEM.

**Figure S2:** Normalized electric-field intensity $\mid E\mid^{2}$ for a PMMA ridge waveguide (width 300 nm, height 50 nm) on a 140 nm Au film including a 0.5 µm-long gap. A pronounced local field-intensity enhancement forms at the gap position, consistent with an increased photoelectron yield in PEEM due to near-field-driven photoemission.

### References

[1]	Flexcompute Inc., *Tidy3D: Electromagnetic Solver for FDTD*. (2025). Zugegriffen: 31. Oktober 2025. [Online]. Verfügbar unter: https://www.flexcompute.com/tidy3d
